# Digital Control Analysis and Design of a Field-Sensed Magnetic Suspension System

**DOI:** 10.3390/s150306174

**Published:** 2015-03-13

**Authors:** Jen-Hsing Li, Juing-Shian Chiou

**Affiliations:** 1 Department of Electrical Engineering, Kun Shan University, 195 Kunda Road, Yongkang District, Tainan City 710, Taiwan; E-Mail: ljh0906@mail.ksu.edu.tw; 2 Department of Electrical Engineering, Southern Taiwan University of Science and Technology, 1 Nan Ti Street, Yongkang District, Tainan City 710, Taiwan

**Keywords:** magnetic field sensor, magnetic suspension system, digital model, output feedback control, state feedback control, mixed LQR/H∞ control

## Abstract

Magnetic suspension systems are mechatronic systems and crucial in several engineering applications, such as the levitation of high-speed trains, frictionless bearings, and wind tunnels. Magnetic suspension systems are nonlinear and unstable systems; therefore, they are suitable educational benchmarks for testing various modeling and control methods. This paper presents the digital modeling and control of magnetic suspension systems. First, the magnetic suspension system is stabilized using a digital proportional-derivative controller. Subsequently, the digital model is identified using recursive algorithms. Finally, a digital mixed linear quadratic regulator (LQR)/H∞ control is adopted to stabilize the magnetic suspension system robustly. Simulation examples and a real-world example are provided to demonstrate the practicality of the study results. In this study, a digital magnetic suspension system model was developed and reviewed. In addition, equivalent state and output feedback controls for magnetic suspension systems were developed. Using this method, the controller design for magnetic suspension systems was simplified, which is the novel contribution of this study. In addition, this paper proposes a complete digital controller design procedure for magnetic suspension systems.

## 1. Introduction

Magnetic suspension systems (MSSs) are important in several engineering applications [1,2,3,4], such as the levitation of high-speed Maglev trains [1,2], frictionless bearings [3], and wind tunnels [4]. Maglev trains travel a contactless guideway using magnetic suspension, therefore the friction is reduced and the speed is faster than possible with wheeled trains. The magnetic bearing is a kind of bearing and it has several notable features, being contact-free, abrasion-free, and lubrication-free. Magnetic bearings can adapt to high rotation speed. Wind tunnel experiments can measure the aerodynamic forces and torques applied to the object with a supporting rod. Magnetic suspension and balance system can sustain aerodynamic models stationary in a wind tunnel without a rod. Magnetic suspension technology is one of crucial techniques in those of engineering systems.

Since 1986, MSSs have been the mechatronic subject of an undergraduate project [5] and have been provided as a teaching example in a control engineering textbook [6]. In research on MSSs, the dynamic control is an important subject because the magnetic force is nonlinear and the open loop system is inherently unstable. Many modeling and controls are adopted to stabilize MSSs. Basically, the modeling methods can be summarized in two aspects. One aspect is analog modeling and the other is digital modeling. Numerous studies [1,2,3,4,5,6] have analyzed and designed MSSs by using analog control models. In this study, an MSS was analyzed and designed using a digital control model [7,8]. In addition, a digital model of an MSS and a digital proportional-derivative (PD) control were reviewed, and the equivalent state and output feedback controls of an MSS were developed. Based on the digital model, a linear-quadratic regulator (LQR)/H∞ control was adopted to improve system dynamics and eliminate disturbances.

For an analog modeling viewpoint, a lot of research has been performed on control methods for MSSs. For example, Wong [1] and Kuo [2] used system linearization and phase-lead compensation techniques to control the unstable nonlinear system. Bittar and Sales [3] proposed H_2_ and H∞ controls, which are based on the analysis and design of the frequency domain and the analog case. Zhang *et al.* [4] established the state feedback linearized suspension module control system model. A PID control method was used to stabilize the suspension module control system model.

From the digital modeling viewpoint, [9,10] are focused on digital controls for MSSs. Qin *et al.* [9] dealt with the MSS by use of model predictive control. The ARX model is adopted. Su *et al.* [10] stabilized the MSS by use of Takagi–Sugeno (T-S) fuzzy control. By employing the Euler first-order approximation, they obtained the T–S fuzzy model. In these situations [9,10], initial stable conditions are important and necessary. Hence, the proposed digital model in this paper is newer and more useful for implementation. A hands-on field-sensed magnetic suspension system is provided to demonstrate the practicality of the study results. Complete sensor and drive circuits are provided and analyzed. In this paper, a second-order digital model was developed and a thorough research description is provided from analysis and design to implementation. This paper gives a new solution of MSSs.

Up to now, with the progress in microcomputers, embedded controllers are widely applied to mechatronic systems. With the use of microcomputers, digital modeling and controls are essentially necessary. Weng and Chao [11] investigated sampled-data robust H∞ control of the active suspension system. The controller design was cast into a convex optimization problem with LMI constraints. Vesely *et al.* [12] studied the problem of a robust static output feedback model predictive controller design. The developed control design was illustrated on the model of a double integrator controlled through network control systems. Shi *et al.* [13] designed a H∞ controller for a class of discrete-time Markov jump systems with missing information. Markov jump systems have application in many practical systems, such as in manufacturing, economic, electrical and communication systems. Li *et al.* [14] addressed the state estimation and sliding mode control problems for Markov jump systems with mismatched uncertainties. Wang *et al.* [15] designed a sliding mode I/O feedback linearization controller for speed and current tracking of the permanent magnet synchronous motor. A sliding mode variable structure input-output feedback linearization controller is proposed. Su *et al.* [16,17] dealt with discrete-time Takagi–Sugeno fuzzy systems. The dynamic output feedback controller design of time delay systems was investigated in [16]. The dynamic output feedback Hankel norm controller design of stochastic systems was investigated in [17]. Liu *et al.* [18] proposed adaptive robust controllers for path following of underactuated surface vessels. The hierarchical sliding mode method was designed.

In this paper, a new digital modeling of MSSs is proposed and a complete controller design procedure is also provided. The simulation results and real world experimental results are guaranteed by the proposed scheme. This paper is structured as follows: Section 2 reviews the digital model and digital PD control; Section 3 presents identification methods of the digital model; Section 4 describes the state and output feedback controls; Section 5 reviews the state LQR/H∞ control; Section 6 presents the experiments and results; and Section 7 provides the conclusions.

## 2. Digital Model and Digital PD Control

The digital model and digital PD control of the MSS are reviewed in this section. The general analog model of an MSS is formulated as follows [1,5,6,7,8]:

(1) $$m\frac{d^{2}x}{dt^{2}}=mg-C\frac{i^{2}}{x^{2}}$$

where m is the mass of the controlled object, x is the distance between the electromagnet and the controlled object, g is the gravitational acceleration, C is the force constant, and i is the coil current. In order to avoid confusing definitions of symbols, symbols of this paper are summarized in Table 1. Using a Taylor series expansion [6] and neglecting all higher-order terms yields the following piecewise linearized equation:

(2) $$\frac{d^{2}}{dt^{2}}\Delta x=-\frac{2Ci_{0}}{mx_{o}^{2}}\Delta i+\frac{2Ci_{0}^{2}}{mx_{o}^{3}}\Delta x$$

where $x_{0}$ is the equilibrium position, $i_{0}$ is the bias current, $\Delta x=x-x_{0}$ is the deviation of the distance, $\Delta i=i-i_{0}$ the deviation of the coil current. The equilibrium condition is:

(3) $$i_{0}=x_{0}\sqrt{\frac{mg}{C}}$$

**Table 1:** Symbol summary.

m	the mass of the controlled object
g	the gravitational acceleration
C	the force constant
ρ	the linear factor of the position sensor
x	the distance between the electromagnet and the controlled object
$x_{0}$	the equilibrium position
Δx	$=x-x_{0}$ the deviation of the distance
ΔX(s),ΔX(z)	the Laplace transform and z transform of Δx
i	the coil current
$i_{0}$	the bias current
Δi	$=i-i_{0}$ the deviation of the coil current
ΔI(s),ΔI(z)	the Laplace transform and z transform of Δi
T	the sampling period
β	$=e^{T\sqrt{\frac{2Ci_{0}^{2}}{mx_{o}^{3}}}}$ the derived parameter
σ	$=\sqrt{\frac{C}{2mx_{o}}}$ the derived parameter
$\tilde{\sigma }$	$=\frac{\text{σ}\rho \left({\text{β}}^{2}-1\right)}{\text{β}}$ the derived parameter
$\tilde{\beta }$	$=\text{β}+{\text{β}}^{-1}$ the derived parameter
G(z)	the z transform of G(s)
$G_{C}(z)$	the transfer function of digital PD controller
K	the parameter of controller $G_{C}(z)$
ϕ	the parameter of controller $G_{C}(z)$
$\Delta \tilde{X}(z)$	the output of the Figure 1, z transform of $\Delta \tilde{x}(k)$
R(z)	the reference input of the Figure 1
Q(z)	the characteristic polynomial of the closed-loop system
$x_{1}(k)$,$x_{2}(k)$	state variables of Equation (34)
$K_{1}$,$K_{2}$	output feedback gains of Equation (36)

**Figure 1:**
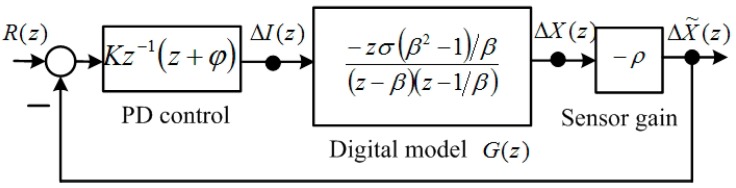
Block diagram of the digital model and PD control.

Applying the Laplace transform of Equation (2) [6] yields the transfer function of an MSS:

(4) $$G(s)=\frac{\Delta X(s)}{\Delta I(s)}=\frac{-\frac{2Ci_{0}}{mx_{o}^{2}}}{s^{2}-\frac{2Ci_{0}^{2}}{mx_{o}^{3}}}$$

where ΔX(s) is the Laplace transform of Δx, and ΔI(s) is the Laplace transform of Δi. The digital model of Equation (4) can be calculated using the residue formula [11]:

(5) $$G(z)=\sum_{\begin{array}{l} \text{at poles}\\ \text{of }G(\text{λ}) \end{array}}\left[\text{residues of }G(\text{λ})\frac{1}{1-z^{-1}e^{T\text{λ}}}\right]$$

where T is the sampling period, and Equation (5) can be expressed as follows:

(6) $$G(z)=\frac{\Delta X(z)}{\Delta I(z)}=\text{σ}\left(\frac{z}{z-\frac{1}{\text{β}}}-\frac{z}{z-\text{β}}\right)=\frac{-z\text{σ}\left({\text{β}}^{2}-1\right)/\text{β}}{(z-\text{β})\left(z-\frac{1}{\text{β}}\right)}$$

The parameters are expressed as follows:

(7) $$\text{β}=e^{T\sqrt{\frac{2Ci_{0}^{2}}{mx_{o}^{3}}}}>1\text{, σ}=\sqrt{\frac{C}{2mx_{o}}}$$

The digital PD control [7,8] is described and formulated as follows:

(8) $$G_{C}(z)=Kz^{-1}(z+\phi )$$

and is adopted, as shown in Figure 1. The term $z^{-1}$ is designed to cancel the z term of G(z) in Equation (6). The value of zero was set to z=−ϕ and the gain K was increased to move the roots into the unit circle. According to Figure 1, the transfer function of $\Delta \tilde{X}(z)/R(z)$ is:

(9) $$\frac{\Delta \tilde{X}(z)}{R(z)}=\frac{\left(z+\phi \right)K\tilde{\sigma }}{\left(z-\text{β}\right)\left(z-1/\text{β}\right)+\left(z+\phi \right)K\tilde{\sigma }}$$

The parameter $\tilde{\sigma }=\sigma \rho \left(\beta^{2}-1\right)/\beta$, where ρ is the linear factor of the position sensor, is a positive real number. The term R(z) is the reference input of the closed-loop system. The characteristic polynomial of the closed-loop system is:

(10) $$Q(z)=z^{2}+z\left(K\tilde{\sigma }-\tilde{\beta }\right)+1+K\tilde{\sigma }\phi$$

where the parameter $\tilde{\beta }={\text{β+β}}^{-1}$. According to the Jury stability criterion [19], the stable conditions for K and ϕ are:

(11) $$\frac{\left(\text{β}-1\right)}{\text{σρ}\left(\text{β}+1\right)\left(1+\phi \right)}<K<\frac{\left(\text{β}+1\right)}{\text{σρ}\left(\text{β}-1\right)\left(1-\phi \right)}$$

and:

(12) $$\left|1+K\tilde{\sigma }\phi \right|<1$$

The inequality in Equation (12) implies that:

(13) $$\frac{-2}{K\tilde{\sigma }}<\phi <0$$

because $\tilde{\sigma }>0$ and K>0. The digital PD control can then be designed as follows. First, the value of zero is set to z=−ϕ, and then the gain K is increased to ensure the stability of the MSS. If this step is not guaranteed, a different zero can be used.

The total current command of the equilibrium point is the sum of the bias current Equation (3) and Δi. Therefore, the current command is:

(14) $$i(k)=i_{o}(k)+\Delta i(k)=i_{o}(k)+K\Delta \tilde{x}(k)+K\phi \Delta \tilde{x}(k-1)$$

where $i_{o}(k)$ is the bias current of the equilibrium point, Δi(k) is the control current, $\Delta \tilde{x}(k)$ is the present position measurement deviation, and $\Delta \tilde{x}(k-1)$ is the previous position measurement deviation.

## 3. Identification Methods

The identification methods used in the magnetic suspension system are presented in this section. Because the digital model is unstable, the digital PD control was used to stabilize the MSS model. SIMULINK simulations for these two identification methods are provided. The results of both these methods are favorable. In this section, the identification of MSS parameters is examined. In Figure 1, the transfer function from ΔI(z) to $\Delta \tilde{X}(z)$ is:

(15) $$\frac{\Delta \tilde{X}(z)}{\Delta I(z)}=\frac{z\tilde{\sigma }}{z^{2}-\tilde{\beta }z+1}$$

The input/output equation is:

(16) $$\Delta \tilde{x}(k)-\tilde{\beta }\Delta \tilde{x}(k-1)+\Delta \tilde{x}(k-2)=\tilde{\sigma }\Delta i(k-1)$$

Equation (16) can then be rewritten as follows:

(17) $$\Delta \tilde{x}(k)+\Delta \tilde{x}(k-2)=\tilde{\beta }\Delta \tilde{x}(k-1)+\tilde{\sigma }\Delta i(k-1)$$

Let the following equations be defined as follows:

(18) $$y(k)=\Delta \tilde{x}(k)+\Delta \tilde{x}(k-2)$$

(19) $$\varphi^{T}(k)=\left[\begin{array}{cc} \Delta \tilde{x}(k-1) & \Delta i(k-1) \end{array}\right]=\left[\begin{array}{cc} \varphi_{1}(k) & \varphi_{2}(k) \end{array}\right]$$

(20) $${\text{θ}}^{T}=\left[\begin{array}{cc} \tilde{\beta } & \tilde{\sigma } \end{array}\right]=\left[\begin{array}{cc} {\text{θ}}_{1} & {\text{θ}}_{2} \end{array}\right]$$

Then the following regression model holds:

(21) $$y(k)=\varphi^{T}(k)\text{θ}$$

The following MSS system parameters are used to minimize the least-squares function as follows:

(22) $$V\left(\text{θ},k\right)=\frac{1}{2}\sum_{n=1}^{k}{\text{η}}^{k-n}{\left(y(n)-\varphi^{T}(n)\text{θ}\right)}^{2}$$

where η is a parameter such that 0<η≤1. The parameter η is called the forgetting factor. Because the measurement outputs are obtained sequentially, the computation time for recursive computations can be reduced. Let $\hat{\theta }(k)$ denote the least-squares estimate based on k’s measurement. The following theorem was developed by Astrom and Wittenmark [20] and is used to estimate system parameters.

**Theorem 1.** *Assume that the matrix* Φ(k) *has full rank. The parameter* θ, *which minimizes Equation (22), is given recursively by:*

(23) $$\hat{\theta }(k)=\hat{\theta }(k-1)+K(k)\left(y(k)-\varphi^{T}(k)\hat{\theta }(k-1)\right)$$

(24) $$K(k)=P(k)\varphi (t)=P(k-1)\varphi (k){\left(\text{η}I+\varphi^{T}(k)P(k-1)\varphi (k)\right)}^{-1}$$

(25) $$P(k)=\left(I-K(k)\varphi^{T}(k)\right)P(k-1)/\text{η}$$

*where* $\Phi^{T}(k)=\left[\begin{array}{ccc} \varphi (1) & \cdots & \varphi (k) \end{array}\right]$ *and* $P(k)={\left(\Phi^{T}(k)\Phi (k)\right)}^{-1}={\left(\sum_{j=1}^{k}\varphi (j)\varphi^{T}(j)\right)}^{-1}$.

A numerical example is provided as follows. In [5], an undergraduate MSS project was described. The parameters measured in [5] are listed in Table 2. The data in Table 2 indicate that the open-loop digital model of an MSS is formulated as follows:

(26) $$G(z)=\frac{\Delta X(z)}{\Delta I(z)}=\frac{-0.0258z}{(z-1.0508)\left(z-0.9517\right)}$$

The parameters in Equation (7) are β=1.0508 and σ=0.2606. If the zero of the digital PD control is defined as ϕ=−0.8 in Equation (8), the stable range of gain is:

(27) $$4.166\times 10^{-4}<K<0.0755$$

If the gain is defined as K = 0.05, the characteristic polynomial of the closed-loop system is:

(28) $$Q(z)=z^{2}-0.5306z-0.1774$$

**Figure 2:**
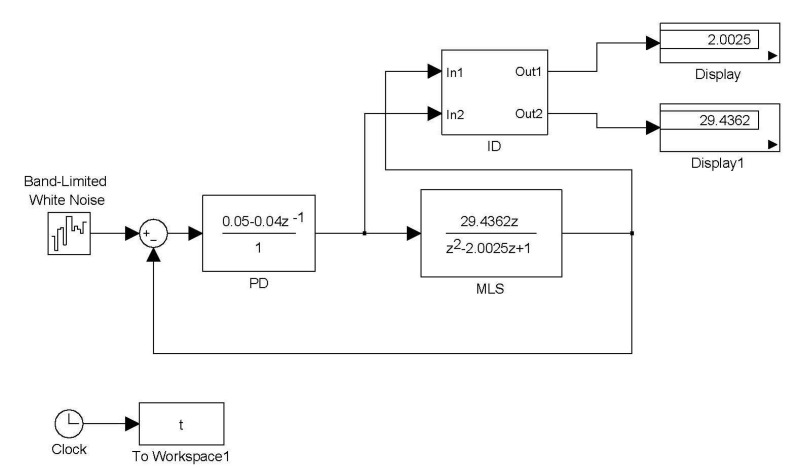
The Simulink simulation of identification.

The eigenvalues of Equation (28) are 0.7632 and −0.2325. All of these poles are inside the unit circle; hence, the closed-loop system is stable. A SIMULINK simulation is shown in Figure 2. The digital PD control Equation (8) is $G_{C}(z)=0.05-0.04z^{-1}$. The digital model of MSS (15) is $29.4362z/(z^{2}-2.0025z+1)$. The command input is white noise. The block ID is the algorithm of Theorem 1. The inputs of the block ID are Δi(k) and $\Delta \tilde{x}(k)$. The outputs of the block ID are estimates of $\tilde{\beta }$ and $\tilde{\sigma }$. The parameter η is 0.75. The results are shown in Figure 2, which are ${\hat{\theta }}_{1}=2.0025$ and ${\tilde{\theta }}_{2}=29.4362$. These results exactly match the parameters of the MSS.

**Table 2:** Measured data of the project in [1].

m	0.068Kg
g	$9.8\frac{m}{s^{2}}$
C	$7.39\times 10^{-5}N\cdot \frac{m^{2}}{A^{2}}$
ρ	$1.14\times 10^{3}\frac{V}{m}$
$x_{0}$	0.008m
$i_{0}$	0.76A

For large dimensions, the majority of computing effort is expended on updating the matrix P. The Kaczmarz algorithm [20] is a simple method for reducing computing effort. However, the convergence rate of this method is slower. The least-squares function Equation (22) is replaced by the following function:

(29) $$V\left(\hat{\theta },k\right)=\frac{1}{2}{\left(\hat{\theta }(k)-\hat{\theta }(k-1)\right)}^{T}\left(\hat{\theta }(k)-\hat{\theta }(k-1)\right)+\bar{\alpha }\left(y(k)-\varphi^{T}(k)\hat{\theta }(k)\right)$$

where $\bar{\alpha }$ is a Lagrangian multiplier. By taking the derivatives with respect to $\hat{\theta }(k)$ and $\bar{\alpha }$, the following equations are obtained:

(30) $$\hat{\theta }(k)-\hat{\theta }(k-1)-\bar{\alpha }\phi (k)=0$$

(31) $$y(k)-\varphi^{T}(k)\hat{\theta }(k)=0$$

The following equation is then produced by combining Equations (30) and (31):

(32) $$\hat{\theta }(k)=\hat{\theta }(k-1)+\frac{\varphi (k)}{\varphi^{T}(k)\varphi (k)}\left(y(k)-\varphi^{T}(k)\hat{\theta }(k-1)\right)$$

**Figure 3:**
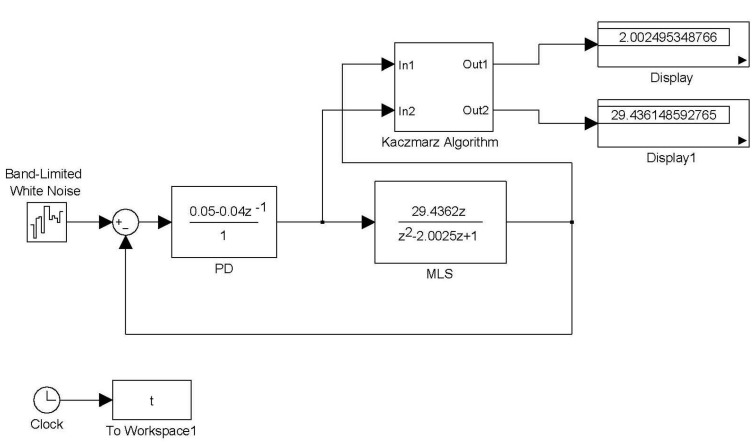
The Simulink simulation of Kaczmarz’s algorithm.

Changing the step size of Equation (32) is useful for avoiding a situation in which φ(k)=0. The following Kaczmarz algorithm is then obtained:

(33) $$\hat{\theta }(k)=\hat{\theta }(k-1)+\frac{\text{μ}\varphi (k)}{\text{α}+\varphi^{T}(k)\varphi (k)}\left(y(k)-\varphi^{T}(k)\hat{\theta }(k-1)\right)$$

where α≥0 and 0<μ<2. The Simulink simulation of the Kaczmarz algorithm is shown in Figure 3. The block Kaczmarz algorithm is the algorithm shown in Equation (33). The step size is μ=1 and the parameter is α=1. The results are ${\hat{\theta }}_{1}=2.002495348766$ and ${\tilde{\theta }}_{2}=29.436148592765$. These results are almost the same as the MSS parameters.

## 4. State and Output Feedback Control

**Figure 4:**
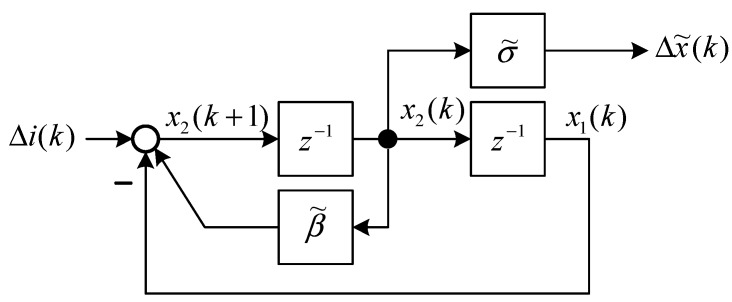
Block model representation of Equations (34) and (35).

**Figure 5:**
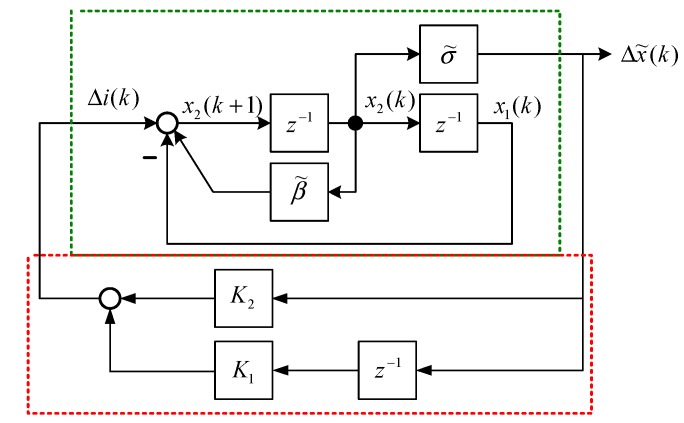
Output feedback control of a MSS.

**Figure 6:**
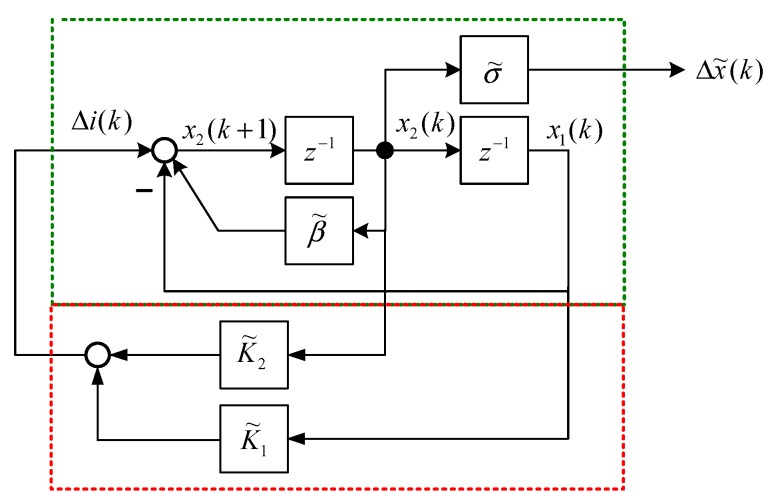
Equivalent state feedback control of a MSS.

The state block diagram of Equation (15) is shown in Figure 4, and the state space representation [19] is expressed as follows:

(34) $$\left[\begin{array}{c} x_{1}(k+1)\\ x_{2}(k+1) \end{array}\right]=\left[\begin{array}{cc} 0 & 1\\ -1 & \tilde{\beta } \end{array}\right]\left[\begin{array}{c} x_{1}(k)\\ x_{2}(k) \end{array}\right]+\left[\begin{array}{c} 0\\ 1 \end{array}\right]\Delta i(k)$$

(35) $$\Delta \tilde{x}(k)=\left[\begin{array}{cc} 0 & \tilde{\sigma } \end{array}\right]\left[\begin{array}{c} x_{1}(k)\\ x_{2}(k) \end{array}\right]$$

where $x_{1}(k)$ and $x_{2}(k)$ are state variables of Figure 4. The output feedback control is shown in Figure 5 and is formulated as follows:

(36) $$\Delta i(k)=K_{2}\Delta \tilde{x}(k)+K_{1}\Delta \tilde{x}(k-1)=K_{2}\tilde{\sigma }x_{2}(k)+K_{1}\tilde{\sigma }x_{1}(k)={\tilde{K}}_{2}x_{2}(k)+{\tilde{K}}_{1}x_{1}(k)$$

where $K_{1}$ and $K_{2}$ are output feedback gains of Figure 5. According to Equation (36), the dynamic output feedback control in Figure 5 is equivalent to the state feedback control in Figure 6. The relationship is expressed as follows:

(37) $$K_{1}=\frac{{\tilde{K}}_{1}}{\tilde{\sigma }},K_{2}=\frac{{\tilde{K}}_{2}}{\tilde{\sigma }}$$

where $K_{1}$ and $K_{2}$ are the control gains of the dynamic output feedback control, and ${\tilde{K}}_{1}$ and ${\tilde{K}}_{2}$ are the control gains of the state feedback control. Figure 6 indicates that the closed-loop system is:

(38) $$\left[\begin{array}{c} x_{1}(k+1)\\ x_{2}(k+1) \end{array}\right]=\left[\begin{array}{cc} 0 & 1\\ -1+{\tilde{K}}_{1} & \tilde{\beta }+{\tilde{K}}_{2} \end{array}\right]\left[\begin{array}{c} x_{1}(k)\\ x_{2}(k) \end{array}\right]$$

The characteristic polynomial of the closed-loop system is:

(39) $$Q(z)=z^{2}-\left(\tilde{\beta }+{\tilde{K}}_{2}\right)z+\left(1-{\tilde{K}}_{1}\right)$$

Comparing the characteristic polynomial of the digital PD control Equations (10) with (39) indicates that the parameters for the control are:

(40) $${\tilde{K}}_{2}=-K\tilde{\sigma },{\tilde{K}}_{1}=-K\phi \tilde{\sigma }$$

and:

(41) $$K_{2}=-K,K_{1}=-K\phi$$

Hence, for the digital model of the MSS, the digital PD control, state feedback control, and output feedback control are nearly the same. First, the state feedback control can be designed. Subsequently, the digital PD control can replace the state feedback control in Equations (40) and (41).

## 5. State Feedback LQR/H∞ Control

This section reviews the state-feedback control and mixed LQR/H∞ control [21]. The digital linear system is:

(42) $$x(k+1)=Ax(k)+B_{1}w(k)+B_{2}u(k)$$

(43) $$z(k)=C_{1}x(k)+D_{12}u(k)$$

where the state vector is $x(k)\in R^{n}$, the control input vector is $u(k)\in R^{m}$, the disturbance vector is $w(k)\in R^{q}$ and belongs to $L_{2}\left[0,\infty \right]$, and the controlled output vector is $z(k)\in R^{p}$; A, $B_{1}$, $B_{2}$, $C_{1}$, and $D_{12}$ are known matrices of the appropriate dimensions. The initial condition is x(0).

The state feedback control is:

(44) u(k)=Fx(k)

where F is a gain matrix with appropriate dimensions. The closed-loop transfer matrix from the disturbance input *w* to the controlled output *z* is

(45) $$T_{zw}(z)=\left[\begin{array}{cc} A_{F} & B_{F}\\ C_{F} & 0 \end{array}\right]=C_{F}{\left(zI-A_{F}\right)}^{-1}B_{F}$$

where $A_{F}=A+B_{2}F$, $B_{F}=B_{1}$, and $C_{F}=C_{1}+D_{12}F$.

The quadratic performance index included in optimal LQR control theory is shown as follows:

(46) $$J=\sum_{k=0}^{\infty }\left(x^{T}(k)Qx(k)+u^{T}(k)Ru(k)\right)$$

where the weighting matrices are Q≥0 and R>0. The ${\text{H}}_{\infty }$ norm is defined for a stable transfer matrix $T_{zw}\left(z\right)$ as follows:

(47) $${\left\|T_{zw}\left(z\right)\right\|}_{\infty }=\underset{w\in [0,2\pi ]}{\sup}{\text{σ}}_{\max}\left\{T_{zw}\left(e^{jw}\right)\right\}$$

where the symbol ${\text{σ}}_{\max}\left\{•\right\}$ denotes the highest singular value.

The state feedback and optimal LQR controls are used to identify an admissible control that minimizes the quadratic performance index Equation (46) under the condition of *w* = 0. However, when using state feedback and ${\text{H}}_{\infty }$ controls, identifying an admissible control such that $\left\|T_{zw}\left(z\right)\right\|\infty <\upsilon$ for a given positive number υ is difficult. In the LQR/H∞ control, these two problems are combined as $w\in L_{2}[0,\infty )$.

The design objective of the state-feedback control and mixed LQR/H∞ control is to identify an admissible control F that minimizes:

(48) $$\underset{w\in L_{2+}}{\sup}\underset{F}{\inf}\left\{J\right\}\text{subject to}{\left\|T_{zw}\left(z\right)\right\|}_{\infty }<\text{υ}$$

To determine the solution, the following assumptions are defined:

Assumption 1: $\left(C_{1},A\right)$ is detectable.

Assumption 2: $\left(A,B_{2}\right)$ is stabilizable.

Assumption 3: $D_{12}^{T}\left[\begin{array}{cc} C_{1} & D_{12} \end{array}\right]=\left[\begin{array}{cc} 0 & I \end{array}\right]$

To determine the solution, the Riccati equation must be solved as follows:

(49) $$A^{T}X_{\infty }A-X_{\infty }-A^{T}X_{\infty }\hat{B}{\left({\hat{B}}^{T}X_{\infty }\hat{B}+\hat{R}\right)}^{-1}{\hat{B}}^{T}X_{\infty }A+C_{1}^{T}C_{1}+Q=0$$

where $\hat{B}=\left[\begin{array}{cc} {\text{γ}}^{-1}B_{1} & B_{2} \end{array}\right]$, $\hat{R}=\left[\begin{array}{cc} -I & 0\\ 0 & R+I \end{array}\right]$. Xu [21] proposed the following theorem, which provides the solution to the state-feedback control and mixed LQR/H∞ control problem.

**Theorem 2.** *A state-feedback control and mixed LQR/H∞* *control* u=Fx *exists if the Riccati equation Equation (49) produces a stabilizing solution* $X_{\infty }\geq 0$ *and* $U_{1}=I-{\text{υ}}^{-2}B_{1}^{T}X_{\infty }B_{1}>0$.

The state-feedback control and mixed LQR/H∞ control is formulated as follows:

(50) $$F=-U_{2}^{-1}B_{2}^{T}U_{3}A$$

where $U_{2}=R+I+B_{2}^{T}U_{3}B_{2}$ and $U_{3}=X_{\infty }+{\text{υ}}^{-2}X_{\infty }B_{1}U_{1}^{-1}B_{1}^{T}X_{\infty }$.

The state-feedback control and mixed LQR/H∞ control achieves:

(51) $$\underset{w\in L_{2+}}{\sup}\underset{F}{\inf}\left\{J\right\}=x^{T}(0)\left(X_{\infty }+{\text{υ}}^{-2}X_{w}-X_{z}\right)x(0)\text{subject to}{\left\|T_{zw}\right\|}_{\infty }<\upsilon$$

where ${\hat{A}}_{F}=A_{F}+{\text{υ}}^{-2}B_{F}U_{1}^{-1}B_{F}^{T}X_{\infty }A_{F}$, $X_{w}=\sum_{k=0}^{\infty }\left\{{\left({\hat{A}}_{F}^{k}\right)}^{T}A_{F}^{T}X_{\infty }B_{F}U_{1}^{-2}B_{F}^{T}X_{\infty }A_{F}{\hat{A}}_{F}^{k}\right\}$ and $X_{z}=\sum_{k=0}^{\infty }\left\{{\left({\hat{A}}_{F}^{k}\right)}^{T}C_{F}^{T}C_{F}{\hat{A}}_{F}^{k}\right\}$.

The numerical example in Section 3 was used to describe the mixed LQR/H∞ control in this section. According to Equations (34) and (35), the state equation and the output equation of the open-loop system are expressed as follows:

(52) $$\left[\begin{array}{c} x_{1}(k+1)\\ x_{2}(k+1) \end{array}\right]=\left[\begin{array}{cc} 0 & 1\\ -1 & 2.0025 \end{array}\right]\left[\begin{array}{c} x_{1}(k)\\ x_{2}(k) \end{array}\right]+\left[\begin{array}{c} 0\\ 1 \end{array}\right]\Delta i(k)$$

(53) $$\Delta \tilde{x}(k)=\left[\begin{array}{cc} 0 & 29.4362 \end{array}\right]\left[\begin{array}{c} x_{1}(k)\\ x_{2}(k) \end{array}\right]$$

The state-feedback control and mixed LQR/H∞ control were then designed. Based on Equations (42) and (43), the system matrices are:

(54) $$A=\left[\begin{array}{cc} 0 & 1\\ -1 & 2.0025 \end{array}\right],B_{2}=\left[\begin{array}{c} 0\\ 1 \end{array}\right],B_{1}=\left[\begin{array}{cc} 1 & 0\\ 0 & 1 \end{array}\right]$$

(55) $$C_{1}=\left[\begin{array}{cc} 1 & 0\\ 0 & 1\\ 0 & 0 \end{array}\right],D_{12}=\left[\begin{array}{c} 0\\ 0\\ 1 \end{array}\right]$$

**Figure 7:**
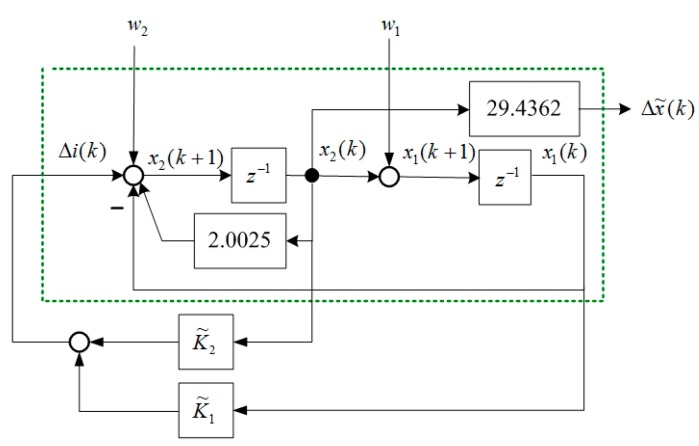
The closed loop system with disturbances.

The closed-loop system with disturbances $w={\left[\begin{array}{cc} w_{1} & w_{2} \end{array}\right]}^{T}$ is shown in Figure 7. The performance variable vector is:

(56) $$z(k)={\left[\begin{array}{ccc} x_{1}(k) & x_{2}(k) & u(k) \end{array}\right]}^{T}$$

MATLAB was used to confirm that Assumption 1 holds because *rank(ctrb(A,B_2_)) = 2* and that Assumption 2 holds because *rank(obsv(A,C_1_)) = 2*. In addition, Assumption 3 holds because the following equation holds:

(57) $$D_{12}^{T}\left[\begin{array}{cc} C_{1} & D_{12} \end{array}\right]=\left[\begin{array}{ccc} 0 & 0 & 1 \end{array}\right]$$

Letting $Q=I_{2}$, R=1, and υ=5, the Riccati equation Equation (49) is solved using the MATLAB command *dare*. The solution is shown as follows:

(58) $$X_{\infty }=\left[\begin{array}{cc} 3.8099 & -3.0264\\ -3.0264 & 10.3759 \end{array}\right]$$

The following matrices are then calculated:

(59) $$U_{1}=I-{\text{υ}}^{-2}B_{1}^{T}X_{\infty }B_{1}=\left[\begin{array}{cc} 0.8476 & 0.1211\\ 0.1211 & 0.5850 \end{array}\right]>0$$

(60) $$U_{3}=X_{\infty }+{\text{υ}}^{-2}X_{\infty }B_{1}U_{1}^{-1}B_{1}^{T}X_{\infty }=\left[\begin{array}{cc} 5.3932 & -6.2897\\ -6.2897 & 19.0393 \end{array}\right]$$

(61) $$U_{2}=R+I+B_{2}^{T}U_{3}B_{2}=21.0393$$

Next, the state feedback gain is calculated as follows:

(61) $$F=-U_{2}^{-1}B_{2}^{T}U_{3}A=\left[\begin{array}{cc} 0.9049 & -1.5132 \end{array}\right]$$.

The poles of the closed-loop system were calculated using the MATLAB command eig(A+B_2_*F), and the eigenvalues were 0.2447±j0.1876. Hence, the closed-loop system was stable.

**Figure 8:**
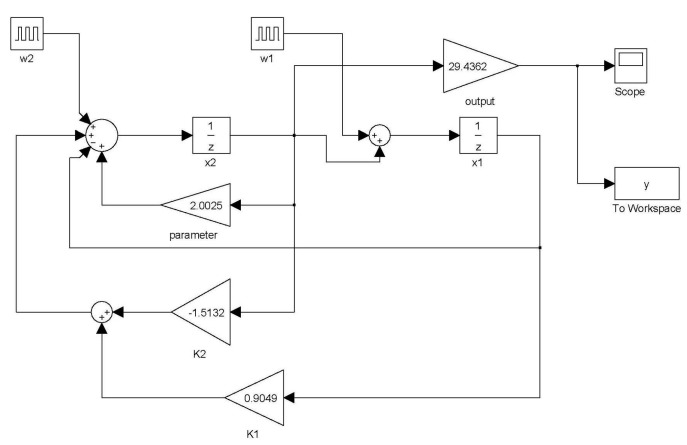
SIMULINK simulation of the closed loop system.

**Figure 9:**
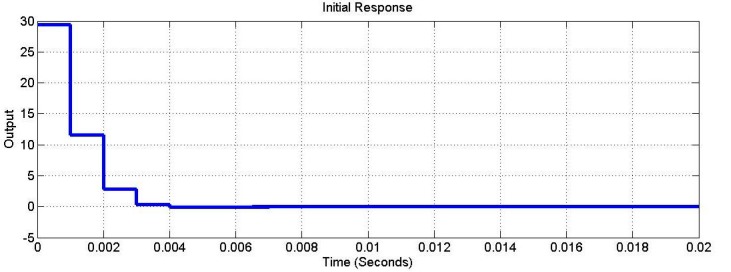
The initial response.

**Figure 10:**
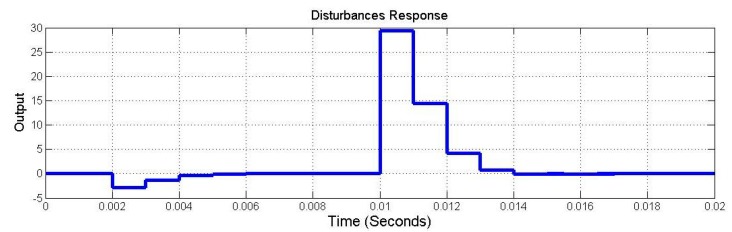
The distrubance response.

The dynamics of the closed-loop system were simulated using SIMULINK (Figure 8). Figure 9 shows the output response under the initial conditions of $x_{1}(0)=1$ and $x_{2}(0)=1$. Figure 10 shows the output response given the disturbances $w_{1}=\text{δ}(t=0)$ and $w_{2}=\text{δ}(t=0.01)$. The symbol $\text{δ}(t=t_{1})$ is the unit impulse function at time $t_{1}$. As shown in Figure 9, the initial condition response was asymptotically stable. As shown in Figure 10, the disturbance $w_{1}=\text{δ}(t=0)$ was added to the closed-loop system and was attenuated to zero at time 0.007 s. The disturbance $w_{2}=\text{δ}(t=0.01)$ was added to the closed-loop system and was attenuated to zero at time 0.018 s. Hence, the disturbances were rejected asymptotically. Based on the simulation results, this control can simultaneously provide optimal output and reject disturbances.

## 6. Experiments and Results

**Figure 11:**
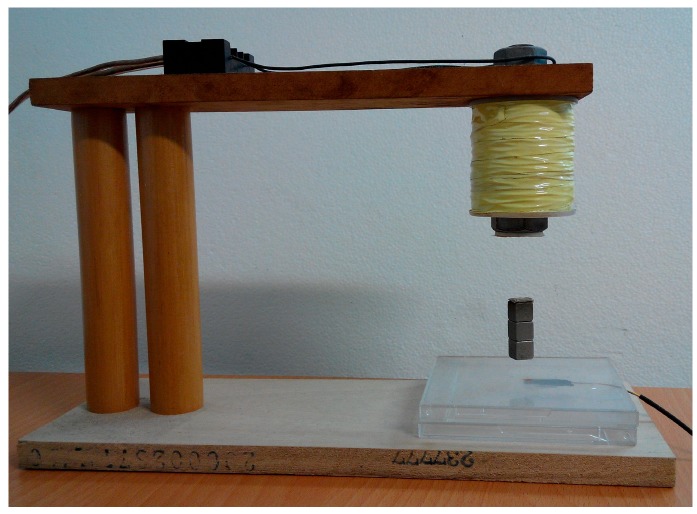
The photograph of a FSMSS.

**Figure 12:**
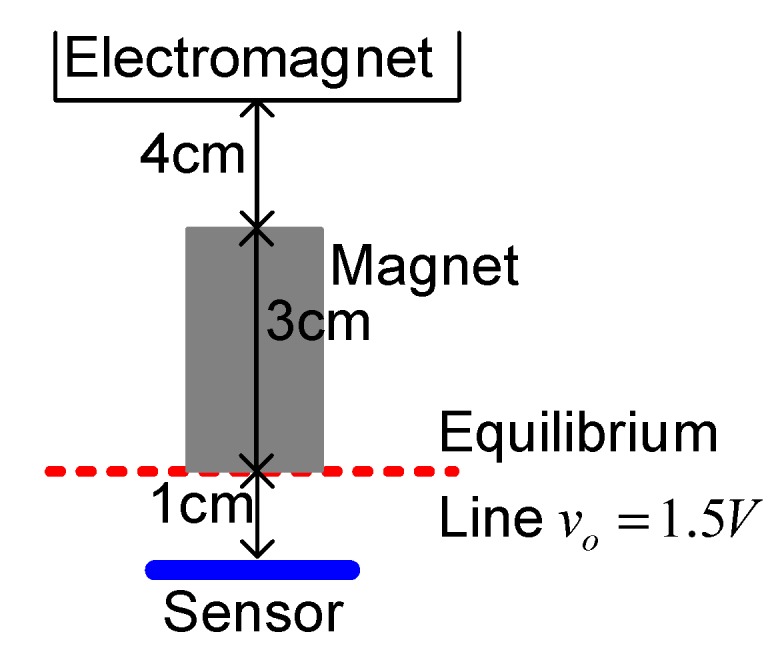
Sketch of the magnet position.

**Figure 13:**
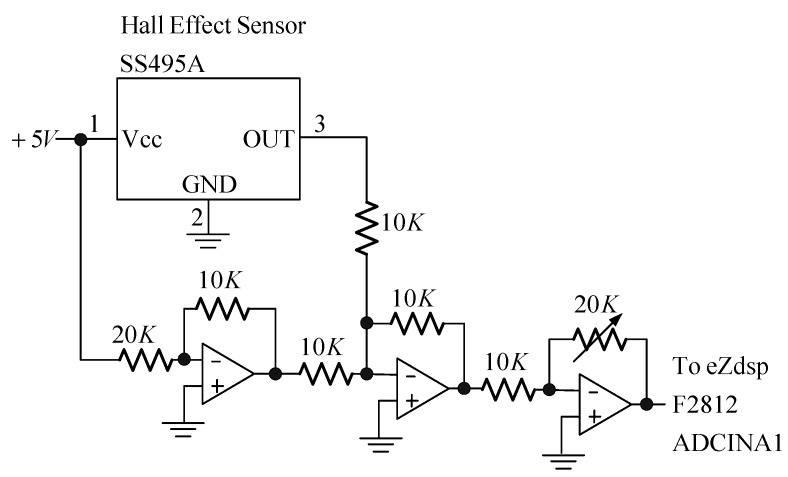
The magnet position sensor circuit.

In this section, the real-world experiment is presented. The setup of the MSS experiment is shown in Figure 11. The position sensor is a Hall element, and it can sense the strength of the magnetic field. Hence, this apparatus is called a field-sensed magnetic suspension system (FSMSS) [8]. A sketch of the magnet position is shown in Figure 12. The magnet is composed of three 1-cm cubes, as shown in Figure 11 and Figure 12. The magnet position sensor circuit is shown in Figure 13. A SS495A magnetic-field sensor was placed at the bottom of the frame. The output measurement varied in proportion to the strength of the magnetic field. An inverting amplifier was used to adjust the magnet position signal within a range of 0–3 V. When the magnet was placed on top of the SS495A, the output voltage was 3 V. As the magnet was pulled away from the SS495A, the output voltage decreased. Thus, the position of the magnet was measured using the circuit shown in Figure 13. The magnet position signal was sampled using the eZdsp F2812 ADINA1 [22]. The sample rate was 1 kHz. As shown in Figure 12, when the bottom of the magnet was exactly *x_o_* = 1 cm away from the sensor, the measured output voltage was 1.5 V. The distance between the electromagnet and the top of the magnet was 4 cm. The bias current required to balance the gravitational force was 0.5 A.

**Figure 14:**
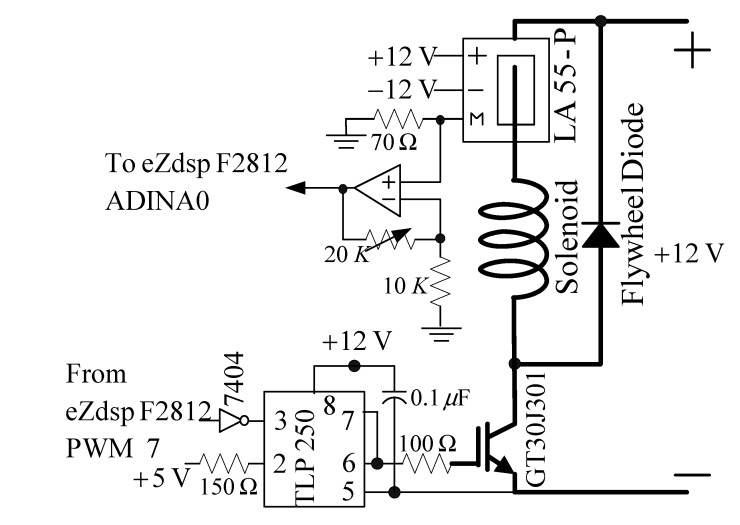
The coil current driver circuit.

The drive circuit shown in Figure 14 included a switch (isolated gate bipolar transistor, IGBT), trigger, and coil-current sensor circuits. A GT30J301 was used as a pulse-width modulation (PWM) switch. The TLP250 used was an IGBT trigger chip. In this PWM circuit, the modulation frequency was 18 kHz. The PWM control was used by the pin PWM 7 of eZdsp F2812 [22]. When the IGBT was off, the flywheel diode could protect the coil current circuit and prevent open-circuit sparking. The LA55-P used was a current sensor. A non-inverting amplifier was used to adjust the coil current signal within a range of 0–3 V. When the coil current was 1 A, the output measurement was 3 V. The coil current signal was sampled using ADINA0 of the eZdsp F2812 [22]. The sample rate was also 1 kHz.

The digital control used was the eZdsp F2812. The 32-bit microcontroller eZdsp F2812 manufactured by Texas Instruments [22] provides motor-control applications. The software pack of the eZdsp F2812 includes the Code Composer Studio (CCS) software. The CCS includes a text editor, a C compiler, an assembler, a debugger, and a download tool. Users can use C programs to implement control theories.

**Figure 15:**
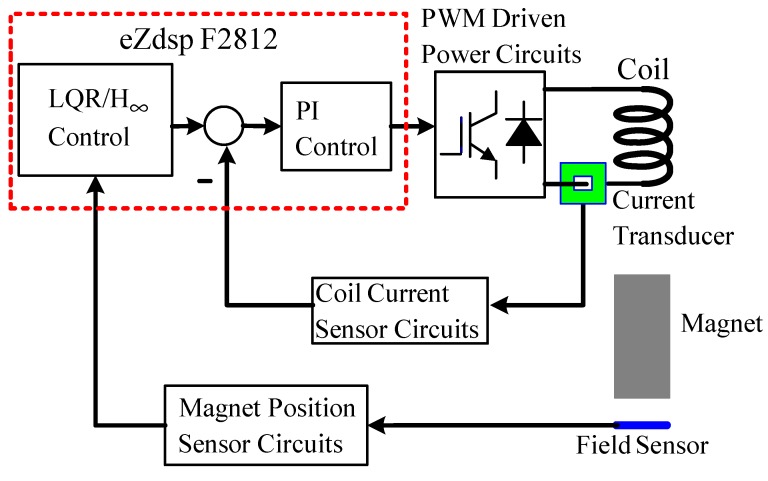
The block diagram of overall system.

**Figure 16:**
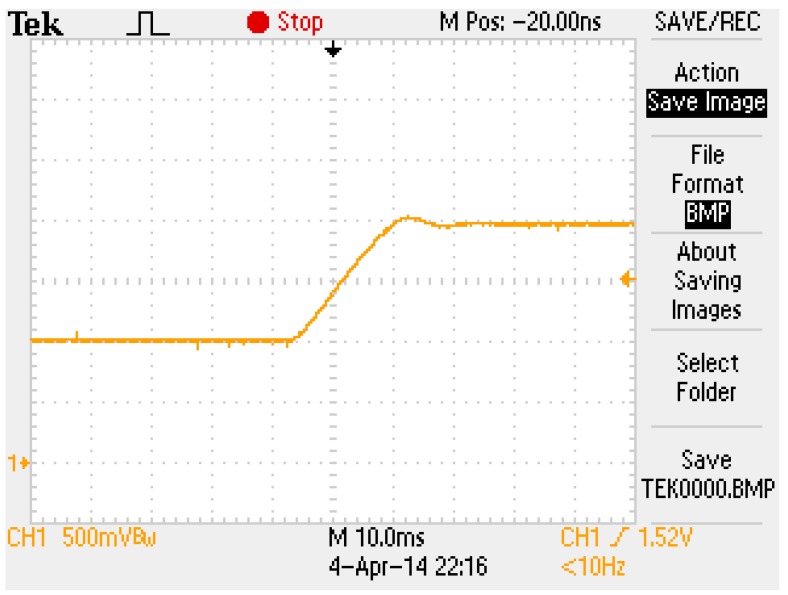
The step response of coil current.

The block diagram of the FSMSS shown in Figure 15 is a two-loop control system. The coil current was compensated in the inner loop and the magnet position was stabilized in the outer loop. First, the coil current was designed using the digital Proportional and Integral (PI) control [7,8]. The PI control can eliminate residual steady-state errors. No steady-state error was observed in this inner-loop subsystem. Figure 16 shows the step response of the coil current signal from 1 to 2 V. In Figure 16, the settling time of the coil current was approximately 25 ms. The dynamics of the coil current were faster than those of the magnet position; therefore, the inner-loop subsystem was approximated as a constant-gain current source. Hence, the overall system can be recognized as a single-input and single-output system. The input was the current command and the output was the magnet position.

**Figure 17:**
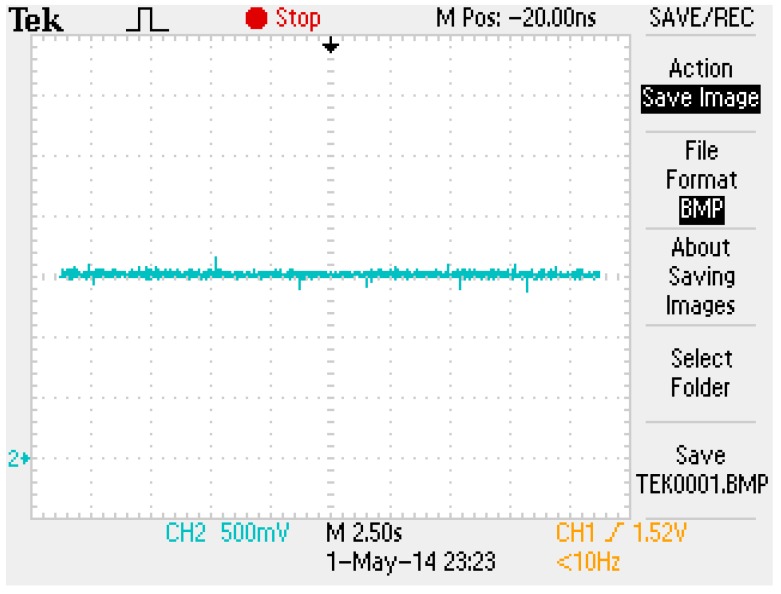
The set-point response of magnet position.

The digital PD control was used to stabilize the FSMSS, as shown in Figure 1. This was a set-point regulation; therefore, the command R(z)=0. The gain of the digital PD control was K=10 and the zero was ϕ=−0.85. The magnet reaches equilibrium at a position measurement of 1.5 V, as shown in Figure 11 and Figure 12. The set-point response is shown in Figure 17. The magnet is stabilized at equilibrium position. Subsequently, the measurement outputs $\Delta \tilde{x}(k)$ and control inputs Δi(k) were recorded in the memory of the microcontroller. The recursive algorithms of the parameters identified in Section 3 were adopted to compute the system parameters $\tilde{\beta }$ and $\tilde{\sigma }$ offline. The results were approximated as follows: $\tilde{\beta }\approx 2.002$ and $\tilde{\sigma }\approx 0.072$.

At the next step, the state-feedback LQR/H∞ control described in Section 5 was used to calculate robust-state feedback gain. The state equation and the output equation of the FSMSS are expressed as follows:

(63) $$\left[\begin{array}{c} x_{1}(k+1)\\ x_{2}(k+1) \end{array}\right]=\left[\begin{array}{cc} 0 & 1\\ -1 & 2.002 \end{array}\right]\left[\begin{array}{c} x_{1}(k)\\ x_{2}(k) \end{array}\right]+\left[\begin{array}{c} 0\\ 1 \end{array}\right]\Delta i(k)$$

(64) $$\Delta \tilde{x}(k)=\left[\begin{array}{cc} 0 & 0.072 \end{array}\right]\left[\begin{array}{c} x_{1}(k)\\ x_{2}(k) \end{array}\right]$$

Based on Equations (42) and (43), the system matrices of the FSMSS are:

(65) $$A=\left[\begin{array}{cc} 0 & 1\\ -1 & 2.002 \end{array}\right],B_{2}=\left[\begin{array}{c} 0\\ 1 \end{array}\right],B_{1}=\left[\begin{array}{cc} 1 & 0\\ 0 & 1 \end{array}\right]$$

(66) $$C_{1}=\left[\begin{array}{cc} 1 & 0\\ 0 & 1\\ 0 & 0 \end{array}\right],D_{12}=\left[\begin{array}{c} 0\\ 0\\ 1 \end{array}\right]$$

The performance variable vector is defined as follows:

(67) $$z(k)={\left[\begin{array}{ccc} x_{1}(k) & x_{2}(k) & u(k) \end{array}\right]}^{T}$$

Letting $Q=I_{2}$, R=1, and υ=5, the Riccati Equation (49) was solved using the MATLAB command *dare*. The solution is shown as follows:

(68) $$X_{\infty }=\left[\begin{array}{cc} 3.8098 & -3.0254\\ -3.0254 & 10.3731 \end{array}\right]$$

The following matrices were then calculated:

(69) $$U_{1}=I-{\text{υ}}^{-2}B_{1}^{T}X_{\infty }B_{1}=\left[\begin{array}{cc} 0.8476 & 0.1210\\ 0.1210 & 0.5851 \end{array}\right]>0$$

(70) $$U_{3}=X_{\infty }+{\text{υ}}^{-2}X_{\infty }B_{1}U_{1}^{-1}B_{1}^{T}X_{\infty }=\left[\begin{array}{cc} 5.3922 & -6.2862\\ -6.2862 & 19.0296 \end{array}\right]$$

(71) $$U_{2}=R+I+B_{2}^{T}U_{3}B_{2}=21.0296$$

Next, the state feedback gain was calculated as follows:

(72) $$F=-U_{2}^{-1}B_{2}^{T}U_{3}A=\left[\begin{array}{cc} 0.9049 & -1.5127 \end{array}\right]$$

Based on the information in Section 4, the relation between state feedback gain and the digital PD control is:

(73) $$\phi =\frac{{\tilde{K}}_{1}}{{\tilde{K}}_{2}}=\frac{-0.9049}{1.5127}\approx -0.6$$

and:

(74) $$K=-K_{2}=\frac{{\tilde{K}}_{2}}{-\tilde{\sigma }}=\frac{-1.5127}{-0.072}\approx 21$$

**Figure 18:**
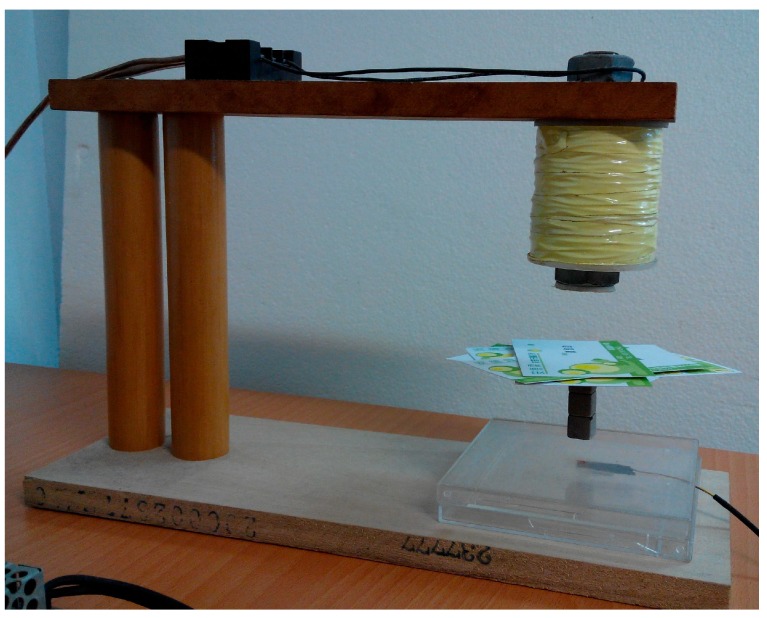
The picture of the FSMSS with six cards.

**Figure 19:**
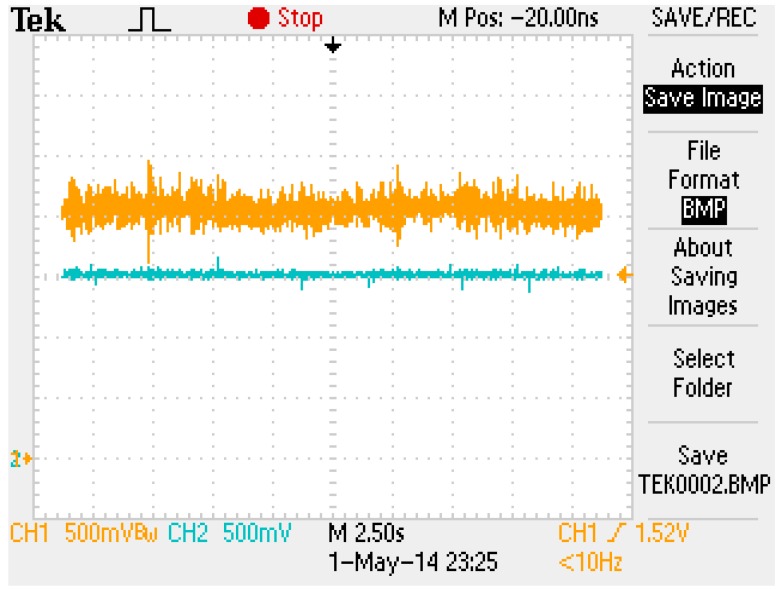
The set-point responses of both magnet position and coil current with six cards.

**Figure 20:**
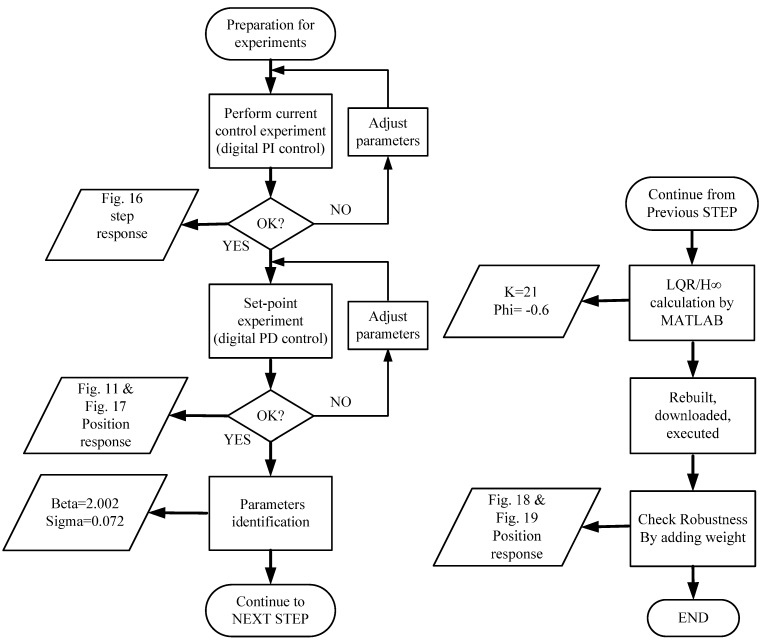
A flowchart of the complete procedure for experiments and results.

The data from Equation (73) and (74) could replace the eZdsp F2812 in the C program. The gain of the digital PD control was adjusted by defining gain as K=21 and zero as ϕ=−0.6. After the parameters were modified using the CCS software, the control program was rebuilt, downloaded, and executed. An experiment was executed by placing six cards on the top of the magnet to demonstrate the robustness. Figure 18 shows the results of this experiment. Figure 19 shows that the magnet position measurement was maintained at 1.5 V and was stable. The yellow line in Figure 19 indicates the coil current measurement. To balance the weight of six cards, the coil current signal increased from 1.5 to 2 V. Based on the results shown in Figure 18 and Figure 19, the stability and robustness of the FSMSS is guaranteed by the mixed LQR/H∞ control. In this section, a complete description of the control design is provided. A flowchart shown in Figure 20 is given to explain clearly the complete procedure for experiments and results.

## 7. Conclusions

The main contribution of this paper is the digital model and control of MSSs. The specified digital PD control was adopted to stabilize the MSS initially. Subsequently, the specified identification methods were developed. Two parameters were identified according to the digital model of MSSs. For designing the robust control, the equivalent state and output feedback controls were developed. By using this equivalence, the control design was simplified. The state-feedback control and mixed LQR/H∞ control were then used to design the robust control. The mixed LQR/H∞ control combines the LQR- and H∞-control designs. This method improves the performance and robustness of the MSS. Numerical examples are provided in this paper and a real-time experimental setup is proposed for demonstrating the use of this method. Thus, this paper provides a complete digital robust control design procedure for MSSs.
